# Comparison of thoracoabdominal versus abdominal-transhiatal surgical approaches in Siewert type II adenocarcinoma at the esophagogastric junction: Protocol for a prospective multicenter randomized controlled trial

**DOI:** 10.3389/fonc.2023.1091615

**Published:** 2023-03-30

**Authors:** Chao Yue, Zhenchang Mo, Xiao Wu, Yannian Wang, Qinchuan Yang, Weidong Wang, Haikun Zhou, Ruiqi Gao, Panpan Ji, Danhong Dong, Ying Zhang, Gang Ji, Xiaohua Li

**Affiliations:** ^1^ Department of Gastrointestinal Surgery, Xijing Hospital, Air Force Medical University, Xi’an, Shaanxi, China; ^2^ Department of Oncology, Affiliated Hospital, Shannxi University of Chinese Medicine, Xianyang, Shannxi, China; ^3^ Department of Radiotherapy, Xijing Hospital, Air Force Medical University, Xi’an, Shaanxi, China

**Keywords:** adenocarcinoma of the esophagogastric junction, Siewert type II, thoracoabdominal, abdominal-transhiatal, surgical approaches

## Abstract

**Background:**

Siewert type II adenocarcinoma of the esophagogastric junction (Siewert II AEG) can be resected by the right thoracoabdominal surgical approach (RTA) or abdominal-transhiatal surgical approach (TH) under minimally invasive conditions. Although both surgical methods achieve complete tumor resection, there is a debate as to whether the former method is superior to or at least noninferior to the latter in terms of surgical safety. Currently, a small number of retrospective studies have compared the two surgical approaches, with inconclusive results. As such, a prospective multicenter randomized controlled trial is necessary to validate the value of RTA (Ivor-Lewis) compared to TH.

**Methods:**

The planned study is a prospective, multicenter, randomized clinical trial. Patients (n=212) with Siewert II AEG that could be resected by either of the above two surgical approaches will be included in this trial and randomized to the RTA group (n=106) or the TH group (n=106). The primary outcome will be 3-year disease-free survival (DFS). The secondary outcomes will include 5-year overall survival (OS), incidence of postoperative complications, postoperative mortality, local recurrence rate, number and location of removed lymph nodes, quality of life (QOL), surgical Apgar score, and duration of the operation. Follow-ups are scheduled every three months for the first 3 years after the surgery and every six months for the next 2 years.

**Discussion:**

Among Siewert II AEG patients with resectable tumors, this is the first prospective, randomized clinical trial comparing the surgical safety of minimally invasive RTA and TH. RTA is hypothesized to provide better digestive tract reconstruction and dissection of mediastinal lymph nodes while maintaining a high quality of life and good postoperative outcome. Moreover, this trial will provide a high level of evidence for the choice of surgical procedures for Siewert II AEG.

**Clinical trial registration:**

Chinese Ethics Committee of Registering Clinical Trials, identifier (ChiECRCT20210635); Clinical Trial.gov, identifier (NCT05356520).

## Background

Adenocarcinoma of the esophagogastric junction (AEG) is defined as a tumor with an epicenter within 5 cm of the esophagogastric junction (EGJ) (1). Over the past two decades, the incidence of AEG has increased significantly, accounting for 5-8% of esophageal cancer in China and 35.7% of gastric cancer and lower esophageal cancer around the world (2–4). Siewert and Stein divided AEGs into three categories based on the distance between the tumor center and the EGJ. The epicenter of a tumor that measures between 1 and 5 cm above the EGJ is defined as type I, while that of an epicenter within 1 cm above and 2 cm below the EGJ is defined as type II, and those within 2–5 cm below the EGJ are defined as type III (5).

Currently, AEG is treated mainly by surgical resection. The National Comprehensive Cancer Network (NCCN) guidelines state that the management of Siewert type I should be similar to that of esophageal cancer, and the thoracoabdominal surgical approach (TA) is recommended because of its higher rate of thoracic lymph node involvement. Siewert type III should be managed as gastric cancer, and an abdominal-transhiatal surgical approach (TH) is recommended (6, 7). However, Siewert type II adenocarcinoma of the esophagogastric junction (Siewert II AEG) differs from the other two types in terms of its anatomical location and biological behavior, which is characterized by high differentiation, deep invasion, susceptibility to metastasis and adverse outcomes (8), making the proper operative approach for Siewert II AEG controversial.

To provide the most effective surgical treatment strategy for patients with Siewert II AEG, the ideal approach should not only consider primary tumor removal and local lymph node dissection but also ensure a safe and effective digestive tract reconstruction method (9). At present, some specialists prefer TA, while others prefer TH (10–12). Compared with TH, TA has a better effect on mediastinal lymph node dissection, with significant advantages for the dissection of subcarinal, paraesophageal, hilar and diaphragmatic lymph nodes (13). And TA provides more operating spaces for resection of the distal esophagus, which ensures a long enough upper resection margin distance from the tumor (10). However, TH can avoid invasion of the chest and minimize pleural complications, which is superior to transthoracic surgery in this respect. In addition, the surgical approach to TA varies in different countries. In Western countries, the right thoracoabdominal surgical approach (RTA) is preferred, whereas in Asian countries, the left thoracoabdominal surgical approach (LTA) is preferred. The results of a prospective study comparing the LTA and RTA showed that the overall number of lymph nodes removed during the LTA was inferior to the RTA, especially for abdominal lymph nodes (14). Further comparison of long-term survival studies showed that the 3-year DFS, OS and local recurrence rate of the RTA were better than those of the LTA (15). Therefore, the NCCN guidelines recommend the RTA rather than LTA for patients with Siewert II AEG (6). Nevertheless, studies have shown that thoracoscopic surgery has a higher incidence of respiratory and cardiovascular complications (16). A meta-analysis including 16 studies indicated that significantly higher incidence of cardiovascular and respiratory complications, and longer length of hospital stay were observed in the RTA group (17). As a result, RTA has certain limitations compared with TH. Thankfully, extensive use of thoracoscopy and laparoscopy in recent years could provide better surgical views and more space for radical surgery of esophagogastric junction Siewert-type adenocarcinoma. A minimally invasive surgical approach can significantly lower the incidence of cardiovascular and respiratory diseases, at the same time reduce intraoperative blood loss, speed up recovery of bowel function and contribute to early discharge compared to open surgery (18). Previous studies have shown that both the minimally invasive Ivor Lewis (RTA) procedure and the minimally invasive abdominal-transhiatal (TH) procedure are superior to open surgery in terms of safety and efficacy for patients with Siewert II AEG (19).

According to the study reported by Wee et al., the minimally invasive RTA approach mainly includes laparoscopic partial or total gastric resection, followed by thoracoscopic distal esophagotomy and mediastinal lymph node dissection (20). The study identified minimally invasive RTA as a viable and safe surgical approach with significantly lower morbidity and mortality compared to open surgery. And Wang et al. reported that minimally invasive TH approach would only be performed by laparoscopy for gastrectomy, distal esophagectomy and diaphragmatic hiatus mediastinal lymph node dissection (19). They believe that minimally invasive TH approach is also a safe and feasible approach with great prospects for clinical application. To date, there are no prospective randomized controlled trials comparing RTA with TH under minimally invasive conditions. Therefore, whether minimally invasive RTA is better than minimally invasive TH is of great research value for the improvement of clinical therapeutic effects for patients with Siewert II AEG.

Based on these points, it is necessary to compare surgical safety, oncology outcomes, and quality of life between RTA and TH for patients with resectable Siewert II AEG in a multicenter randomized controlled trial.

## Methods

### Objective

The purpose of this trial is to compare RTA with TH for resectable Siewert II AEGs based on surgical safety, clinical efficacy and prognosis. The primary outcome of the study will be the 3-year disease-free survival (DFS) to assess the oncological safety of the procedure. The secondary outcomes will be 5-year overall survival (OS), incidence of postoperative complications, postoperative mortality, local recurrence rate, number and location of removed lymph nodes, surgical Apgar score, duration of the operation and the quality of life (QOL) score.

### Study design

This is the first prospective multicenter randomized clinical trial comparing the efficacy of RTA and TH, which will be carried out in China. Centers participating in the trial will include Xi-jing Hospital, Tang-du Hospital, Henan Province People’s Hospital, General Hospital of Ningxia Medical University, First Affiliated Hospital of Xi ‘an Jiaotong University and the First Affiliated Hospital of Shanxi Medical University. The Declaration of Helsinki statement, as well as the international ethical guide to human biomedical research, form the guiding principles of this research. This study has been registered at clinical-trial.gov (NCT05356520) and approved by the Chinese Ethics Committee of Registering Clinical Trials (ChiECRCT20210635). Upon modification of the protocol, the participating institutes will be notified, and the ethics committee will need to approve it again if the change substantially affects the trial. Surgeons who are competent in both approaches will conduct this study. Throughout the study, surgeons, patients, and coordinating researchers will not be blinded to the group allocation.

### Study population

This trial will evaluate patients with Siewert II AEG whose tumors can be safely resected by both minimally invasive RTA approach and minimally invasive TH approach.

### The inclusion criteria for this study are as follows

·Siewert II AEG confirmed histologically
·Both RTA and TH can safely resect the tumor
·Pretreatment stage cT_1-4a_, N_0-3_, M_0_ (referring to the 8th AJCC TNM staging system)
·Aged 18-75
·The Eastern Cooperative Oncology Group (ECOG) performance status ranges from 0 to 2
·American Society of Anesthesiologists (ASA) <4
·Laboratory tests: hemoglobin > 90 g/L, white blood cells > 3×10^9^/L, platelet > 100×10^9^/L, glomerular filtration rate > 60 ml/min, total bilirubin < 1.5x upper level of normal (ULN), aspartate aminotransferase (AST) and alanine aminotransferase (ALT) < 2.5x ULN
·Informed consent is voluntarily signed by the patients and their families

### The exclusion criteria are as follows

·Siewert type I and III adenocarcinoma of the esophagogastric junction confirmed histologically
·A tumor that extends more than five centimeters from the EGJ or has developed distant metastases (M1) or peritoneal invasion
·Significant cardiovascular disease, such as coronary atherosclerosis or myocardial infarction, with symptoms in the past 6 months
·Significant respiratory disease, defined by whether the forced expiratory volume in 1 second (FEV1) is less than 1.5 L/s
·History of right thoracotomy, adhesions to the right pleura or prior epigastric surgery
·Indocyanine green test is not less than 15% for significant cirrhosis or chronic liver disease
·Overt central nervous system disorders, mental disorders, and psychological disorders
·Significant coagulopathy that has not been modified by current technology
·Significant endocrine disorders, such as uncontrolled diabetes
·A disease that is seriously out of control, such as a recurrent infection
·Tumor-related diseases that require emergency surgery due to special conditions such as bleeding, perforation and obstruction

### The termination test standards are as follows

Patients will be terminated if any of the following conditions occur, and the study analysis will not include data from these individuals:

·Patients who are found to be inoperable for a variety of reasons after trial registration (the reasons should be documented in detail)
·The investigator considers the patient unfit for further participation in the study (reasons for withdrawal should be recorded in detail)
·The patient requests termination of the trial
·The patient violates the treatment principles (violating the admission criteria, disobeying the study arrangements, etc.)

### Patient screening

**Figure 1:**
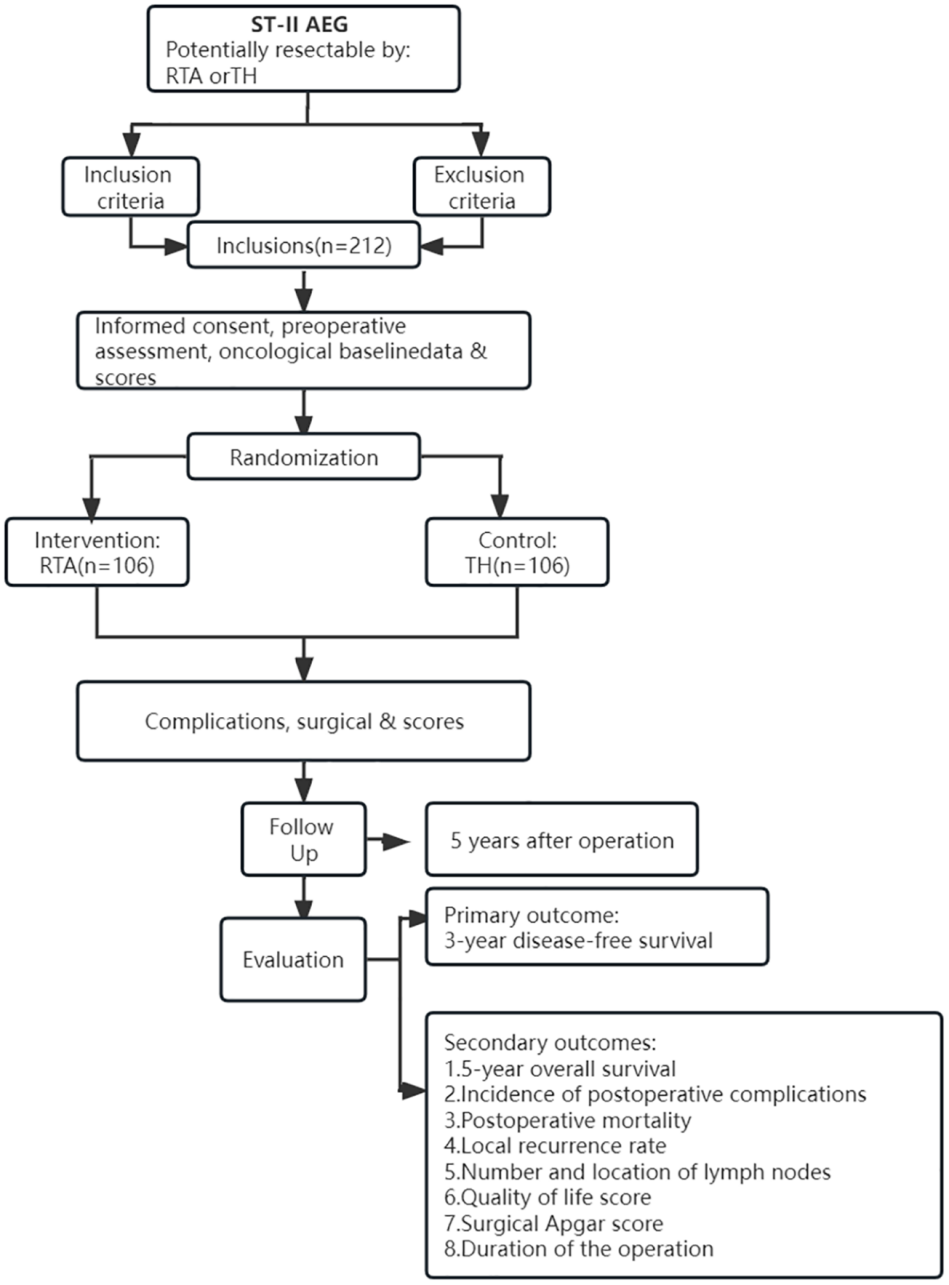
Flow chart.

**Figure 1** displays the trial flow. Before enrolling a patient, a comprehensive assessment will be performed to determine whether the patient meets the enrollment criteria. The results of the endoscopy and pathological analysis will be used to determine whether the classification criteria of Siewert II AEG are met. CT, enhanced CT, MRI, PET-CT and endoscopy will be used to identify and judge the tumor infiltration depth and the possibility of distant metastasis. A physical examination and laboratory tests for the patients are also essential screening methods. In addition, the medical history and basic demographics should be included in the complete preoperative work-up.

### Patient inclusion and randomization

After all of the patients have completed the baseline assessment, they will be reviewed on a case-by-case basis according to the trial’s inclusion and exclusion criteria. Subsequently, an opaque envelope will be unsealed by a research assistant who is not involved in the recruitment and review of patients. This envelope contains a random number table for randomly assigning people who meet the trial requirements to either the RTA or TH group. The patients and surgeons cannot be blinded to their assignment, whereas the pathologists will be blinded to the patient’s group assignment.

### Patient follow-up

According to the schedule (**Table 1**), the patients will be followed for the first time one month after surgery and then every three months for three years. An additional follow-up survey of the indicators will be conducted semiannually for the next two years. All patients will be required to be followed for at least five years. Each follow-up will include a physical examination, a routine blood examination and a serum tumor marker examination every three or six months. Enhanced thoracic and abdominal CT will be performed every six months, and gastroscopy will be performed annually. If a tumor recurrence or metastasis is suspected, additional tests will be performed and recorded on the CRF table. Trial-related complications will be assessed based on the Clavien−Dindo classification. If grade III or above complications occur, they will be reported to the responsible unit of the project, fed back to the study supervision department, and comprehensively evaluated to how to deal with this situation in time and effectively. All follow-up will be performed by the project’s professional follow-up team. The trial will be completed until the last patient completes their follow-up.

**Table 1:** Checklist for collection of necessary clinical data and follow-up schedule of enrolled patients.

	Baseline information				Follow-up (months)																
	Preoperation	Operation	POD 1-7	Discharge	1	3	6	9	12	15	18	21	24	27	30	33	36	42	48	54	60
Demography	√																				
Informed consent	√																				
Medical history	√																				
Oncological history	√																				
Tumor classification	√																				
Biometric data	√																				
Laboratory	√						√		√		√		√		√		√	√	√	√	√
Physical examination	√				√	√	√	√	√	√	√	√	√	√	√	√	√	√	√	√	√
Inclusion / Exclusion	√																				
Randomisation	√																				
Anamnesis	√				√	√	√	√	√	√	√	√	√	√	√	√	√	√	√	√	√
Gastroscopy	√								√				√				√		√		√
Enhanced thoracic and abdominal CT	√						√		√		√		√		√		√	√	√	√	√
Abdominal ultrasound	√						√		√		√		√		√		√	√	√	√	√
Concomitant Medication	√			√	√	√	√	√	√	√	√	√	√	√	√	√	√	√	√	√	√
Surgical information		√																			
Pathology				√																	
postoperative complications			√	√	√																
postoperative mortality			√	√	√																
local recurrence rate				√	√	√	√	√	√	√	√	√	√	√	√	√	√	√	√	√	√
surgical Apgar score		√																			
SF-36	√			√	√	√	√	√	√	√	√	√	√	√	√	√	√	√	√	√	√
EORTC QLQ-C30	√			√	√	√	√	√	√	√	√	√	√	√	√	√	√	√	√	√	√
EORTC QLQ-OES18	√			√	√	√	√	√	√	√	√	√	√	√	√	√	√	√	√	√	√
**Disease-free survival**					√	√	√	√	√	√	√	√	√	√	√	√	√	√	√	√	√
Overall survival					√	√	√	√	√	√	√	√	√	√	√	√	√	√	√	√	√

√, Indicates the need to collect the clinical data

SF-36, Short-Form 36; EORTC QLQ-C30, European Organization for Research and Treatment of Cancer Quality of Life Questionnaire Core 30; EORTC QLQ-OES18, Computerized adaptive test European Organization for Research and Treatment of Cancer Quality of Life Questionnaire for Esophageal cancer.

### Intervention

An equal number of patients will be randomly assigned to the RTA and TH groups. Details of the surgery are determined by the surgeons at each center as long as the tumor can be resected completely. If the tumor is difficult to be resect under minimally invasive conditions, the surgical strategy will be adjusted at that time.

The TH approach will be performed by distal esophagectomy and mediastinal lymph node dissection *via* the diaphragmatic hiatus under laparoscopy, whereas the RTA approach will be performed under thoracoscopy. The resection margin is a key indicator for evaluating the curative effect of surgery. The 5-year mortality of patients with positive resection margins is significantly higher than that of patients with negative resection margins (21).

According to the NCCN guidelines (6) and the Chinese expert consensus, for Siewert type II AEG with cT1 stage, the upper esophageal resection margin is recommended to be at least 2 cm away from the tumor, while for patients with cT2 stage or above, the upper esophageal resection margin is recommended to be at least 5 cm away from the tumor if the RTA approach is performed and at least 3 cm if the TH approach is performed. For patients in the former staging category, the lower resection margin is recommended to be at least 3 cm, while for patients in the latter staging category, a minimum of 5 cm is required to meet the surgical requirements. Laparoscopic proximal gastrectomy can be performed regardless of the surgical approach if the above criteria for the lower resection margin are met and at least more than half of the residual stomach remains.

In addition, total gastrectomy with D2 lymphadenectomy is recommended if the tumor involves more than 4 cm of the stomach. Lower mediastinal lymph node dissection is generally not required when the tumor is less than 2 cm away from the esophagus but is required when it is 2 cm or more. It is important to note that dissection of the upper, middle, and lower mediastinal lymph nodes is recommended once the tumor has invaded the esophagus at a distance of 4 cm or more. Postoperative reconstruction of the digestive tract will be determined by the surgeon’s personal experience and the patient’s situation. Proximal gastrectomy is feasible with gastric tube reconstruction and esophagogastrostomy. Roux-en-Y (esophagojejunostomy and jejunojeju-nostomy) reconstruction is recommended for total gastrectomy. In addition, minimally invasive surgery might need to be converted to open surgery if complications arise during the surgery. And participants may discontinue their participation at any time during the study.

### Surgical quality control

Each center must be a tertiary hospital that has performed at least 20 minimally invasive RTAs and 20 THs in each of the past three years. Surgeons who are skilled in both procedures and have performed each procedure at least 20 times will be eligible for the trial. To ensure surgical quality and facilitate whole-course monitoring, photographs should be taken during each operation to show the integrity of lymph node dissection and tumor resection. If the R0 resection rate is found to be low or the effect of dissected lymph nodes cannot meet requirements during the monitoring process, it will be necessary to analyze the problems and determine the possible reasons.

### Pathological quality control

For the pathological examination to be of high quality, all samples from lymph node stations and peritumoral stations will be examined and analyzed by the pathology department. The surgeon will pack every lymph node station during the lymph node dissection, allowing the pathologist to examine the station individually. In contrast, the peritumoral stations will be marked as a whole rather than individually resected to ensure that the margin of resection can be accurately analyzed. Each center’s lead pathologist will review the slides of ten percent of all cases. Except for the tissue for pathological analysis, which needs to be stored in wax blocks for 30 years, the rest of the tissue samples submitted for examination will be destroyed prior to pathological analysis, while all blood samples will be destroyed after pathological analysis.

### Postoperative treatment

There will be no difference in postoperative treatment between the two groups. Analgesia and antibiotics will be administered according to the standards of each trial site. The surgeons at each participating institution will be responsible for implementing postoperative fluid rehydration and nutritional support. Patients with advanced AEG will routinely receive postoperative adjuvant chemotherapy. Each trial center will discharge patients in accordance with their standard practices.

### Outcome measures

The primary outcome is a comparison of 3-year disease-free survival (DFS) between the two groups. The secondary outcomes of the trial included 5-year overall survival (OS), incidence of postoperative complications, postoperative mortality, local recurrence rate, the number of lymph nodes, quality of life (QOL) score, surgical Apgar score and duration of the operation. All types of postoperative complications will be defined by the Esophageal Complications Consensus Group (ECCG) (22) and classified by the Clavien−Dindo grading system (23). A variety of questionnaires will be used to assess QOL. General health aspects will be measured by the SF-36 and CAT EORTC QLQC30, whereas esophageal health will be assessed by the CAT EORTC QLQ-OES18 (24).

### Data collection and management

The clinical data will be completely, timely, accurately and truthfully recorded in the CRF table by the study coordinator. Any changes that are made will be signed and dated by the person concerned, but these changes will never involve the original data. Since randomization is centralized, each participating center will use thestratified-field block-randomization method (25). The study coordinator randomly assigned study populations meeting inclusion and exclusion criteria to either the RTA group or the TH group based on random numbers drawn from the hidden envelope. And each center will assign another study coordinator to be responsible for the data entry and uploading. The project sponsor or the clinical coordinator on behalf of the sponsor will be in regular contact with the center to provide information and technical support. In this way, the investigators can be supervised to strictly implement the study protocol, and to a certain extent, the accuracy of the clinical information on the CRFs can be verified. Authorized representatives of the project undertaking units, regulatory authorities, and ethics committees have the right to audit and inspect the works of each research center at any time, including original data verification. The purpose of an audit or inspection by the project undertaking unit is a comprehensive and targeted review of all study-related activities and documentation, which can guarantee that these activities are supervised in accordance with the guidelines of the research proposal and other regulatory requirements, and the clinical data are accurately analyzed, recorded and reported.

### Sample size calculation

To calculate the required sample size, the primary outcome is taken into account. According to a previous retrospective study (26), the 3-year disease-free survival (DFS) is 47.6% for RTA and 32% for TH. Then, we calculated 212 patients (106 in the RTA group and 106 in the TH group) need to be enrolled in this trial using a one-sided two-sample t-test. The conventional type I error is 5%, the statistical power is 90%, and the dropout rate is 15%. Prior to participating in the trial, every center should report the number of patients likely to be recruited with reference to the number of patients admitted in recent years for Siewert II AEG.

### Statistical analysis

Standard descriptive statistics will be used to analyze the qualitative and quantitative variables, including the relative and absolute frequencies, means, medians, standard deviations, and interquartile ranges. Continuous variables will be compared using Student’s t-tests or Mann−Whitney U tests, while categorical variables will be compared using chi-square tests or Fisher’s exact tests. A confidence of 95% will be considered suitable for analysis. Statistical significance will be defined as a p value of 0.05 or less. IBM SPSS (Version 28, Chicago, USA) will be used to conduct the statistical analysis.

### Ethical approval and consent to participate

This is a prospective multicenter randomized controlled study aiming to identify the optimal surgical approach for Siewert II AEG. All legal requirements, regulations, and general principles of conduct in human biomedical research will be strictly followed in this study. In addition, the Declaration of Helsinki and the International Code of Ethics for Biomedical Research Involving Humans are essential principles guiding the conduct of this research. Clinical trials registered with the Chinese Ethics Committee have approved the research (Approval No. ChiECRCT20210635, 30 January 2022). The ethics committee is obliged to assess the progress of the research periodically and will be notified in case of any adverse events (AES).

## Discussion

The incidence of adenocarcinoma at the esophagogastric junction (AEG) has increased from 22.3% to 35.7% in the last few decades (4). AEG is highly likely to recur and metastasize, which results in a poor outcome (27). Surgical resection is considered to be the main curative treatment with a favorable prognosis. As the minimally invasive techniques of laparoscopy and thoracoscopy are in widespread use, the choice of surgical methods has become more diverse. Lymph nodes may be dissected by various surgical approaches, which will have a strong impact on the prognosis. However, intense debate has raged for decades regarding the proper operative approach for AEG, especially for Siewert II AEG (8).

The previous literature has shown that the main surgical methods for Siewert II AEG include TH and TA (7). A 10-year follow-up of the JCOG9502 study in Japan showed that LTA was not only ineffective in improving overall or disease-free survival but also increased postoperative morbidity. Therefore, in cases of esophageal invasion depths under 3 cm in AEG type II tumors, they recommended avoiding the LTA approach. However, that study had some limitations, such as a failure to show survival differences between the two procedures, not including minimally invasive procedures, and using only LTA instead of the Ivor-Lewis approach (28).

In recent years, a large number of studies have shown that the LTA approach is inferior to the RTA in terms of the number of lymph nodes dissected, long-term survival, recurrence and prognosis, causing the LTA approach to fall out of favor (6, 14, 15, 29). Compared with the LTA approach, Blank et al. found that the RTA (Ivor-Lewis operation) had a significantly longer survival time than the TH approach. Multivariate analysis showed that the surgical type was an independent prognostic factor. Nevertheless, that study was a single-center, nonrandomized, controlled study that did not use minimally invasive surgery (10). In contrast, a single-center retrospective study found that the TH is more effective in achieving an optimal extent of lymph node dissection, reducing complications, shortening hospital stays, and promoting recovery (11). Although RTA has great advantages in mediastinal lymph node dissection, ensuring a negative esophagectomy margin and completing gastrointestinal reconstruction, it also invades the chest and increases the incidence of chest-related complications (10, 12).

With the introduction of laparoscopic fundoplication in 1991, minimally invasive surgical approaches have been noticed and accepted by a wide range of surgeons and have the potential to reduce surgical morbidity, especially pulmonary complications, promote postoperative recovery and improve the postoperative survival rate, along with leaving only small incisions (25, 30, 31). Therefore, a minimally invasive approach may greatly improve the defects of the TA approach by eliminating its higher postoperative complications. Li KK et al. found that compared with single laparoscopic surgery, a multiple thoracoscopic operation produced little additional trauma to patients and did not increase the incidence of postoperative complications or mortality (32). However, as most of these results are retrospective studies and small in size, their conclusions should be treated with caution.

Currently, no prospective randomized controlled trials have been conducted of minimally invasive surgery for Siewert II AEG. This study will be the first multicenter randomized controlled trial focusing on Siewert II AEG treated with minimally invasive surgery, comparing the clinical efficacy of RTA versus TH. Upon successful completion of this study, we will be able to provide basic information associated with each surgical approach about disease-free survival, overall survival, postoperative complications, tumor outcomes and prognosis. The objective of this trial is to determine whether the RTA approach for Siewert II AEG patients is superior to or at least noninferior to TH in terms of surgical safety. In conclusion, the results of this study will provide clinical guidelines for choosing an approach for Siewert II AEG surgery. We hypothesized that the efficacy of digestion tract reconstruction and dissection of mediastinal lymph nodes by RTA would be better. We predict that when using minimally invasive techniques, the 3-year disease-free survival, 5-year overall survival and other prognostic indicators of RTA will be superior to or at least noninferior to that of TH, while providing a high quality of life and good postoperative outcome.

## Strengths and weaknesses

### Strengths

Among patients with resectable Siewert II AEG, this is the first prospective, randomized controlled trial comparing the efficacy of minimally invasive RTA and TH. Previous studies have not reported a prospective and reliable comparison of postoperative safety between the two procedures.

### Limitations

The study population will be mainly composed of Chinese individuals, and its representativeness has certain limitations.

## Data availability statement

The raw data supporting the conclusions of this article will be made available by the authors, without undue reservation.

## Ethics statement

The studies involving human participants were reviewed and approved by Chinese Ethics Committee of Registering Clinical Trials. The patients/participants provided their written informed consent to participate in this study.

## Author contributions

YZ, GJ and XL developed the design and methodology. CY wrote and drafted this manuscript. ZM has made contributions to the registration of research ethics. XL and CY were responsible for developing the plans for statistical analysis. DD, PJ, RG, QY, WW, YW, HZ and XW will be responsible for the observation and data collection of those enrolled in this trial. The final manuscript will be reviewed and approved by all authors.
